# Utilization of Single-Pulse Transcranial-Evoked Potentials in Neurological and Psychiatric Clinical Practice: A Narrative Review

**DOI:** 10.3390/neurolint16060106

**Published:** 2024-11-11

**Authors:** Hilla Fogel, Noa Zifman, Mark Hallett

**Affiliations:** 1 QuantalX Neuroscience Ltd., Kfar-Saba 4453001, Israel; noazif@gmail.com; 2 National Institute of Neurological Disorders and Stroke, National Institutes of Health, Bethesda, MD 20892, USA; worldneurology@gmail.com

**Keywords:** single-pulse transcranial magnetic stimulation (TMS), TMS-evoked potentials (TEPs), neurophysiologic biomarker, TMS-EEG, Alzheimer’s disease, Parkinson’s disease, major depressive disorder, stroke recovery, treatment response

## Abstract

*Background:* The utility of single-pulse TMS (transcranial magnetic stimulation)-evoked EEG (electroencephalograph) potentials (TEPs) has been extensively studied in the past three decades. TEPs have been shown to provide insights into features of cortical excitability and connectivity, reflecting mechanisms of excitatory/inhibitory balance, in various neurological and psychiatric conditions. In the present study, we sought to review and summarize the most studied neurological and psychiatric clinical indications utilizing single-pulse TEP and describe its promise as an informative novel tool for the evaluation of brain physiology. *Methods:* A thorough search of PubMed, Embase, and Google Scholar for original research utilizing single-pulse TMS-EEG and the measurement of TEP was conducted. Our review focused on the indications and outcomes most clinically relevant, commonly studied, and well-supported scientifically. *Results:* We included a total of 55 publications and summarized them by clinical application. We categorized these publications into seven sub-sections: healthy aging, Alzheimer’s disease (AD), disorders of consciousness (DOCs), stroke rehabilitation and recovery, major depressive disorder (MDD), Parkinson’s disease (PD), as well as prediction and monitoring of treatment response. *Conclusions:* TEP is a useful measurement of mechanisms underlying neuronal networks. It may be utilized in several clinical applications. Its most prominent uses include monitoring of consciousness levels in DOCs, monitoring and prediction of treatment response in MDD, and diagnosis of AD. Additional applications including the monitoring of stroke rehabilitation and recovery, as well as a diagnostic aid for PD, have also shown encouraging results but require further evidence from randomized controlled trials (RCTs).

## 1. Introduction

Combining transcranial magnetic stimulation (TMS) and electroencephalography (EEG) (TMS-EEG) has been shown to have great value as a direct, objective, and non-invasive tool for the evaluation of brain states and dynamics, including cortical excitability, connectivity, plasticity, and excitation–inhibition mechanisms within different brain networks. The combination of TMS-EEG enables direct assessment of cortical circuits. By directly stimulating the brain, this technology bypasses peripheral and cognitively initiated sensory and motor pathways required in event-related potentials (ERPs), sensory-evoked potentials (SEPs), and motor-evoked potentials (MEPs), as TMS-EEG is not reliant on the integrity of these systems or willingness of the patient to participate [1,2].

Accumulating evidence from numerous studies over the past 25 years positions TMS-EEG as a powerful emerging tool in various clinical indications and a potential aid for diagnostic, prognostic, and monitoring purposes. The key promising areas identified include: neurodegenerative disorders (e.g., Alzheimer’s and Parkinson’s disease), disorders of consciousness, psychiatric diseases, stroke rehabilitation, epilepsy, and treatment efficacy [3,4].

TMS-evoked potentials (TEPs) have been shown highly reproducible within individuals over the occipital, parietal, premotor, motor, and prefrontal regions [5,6,7,8].

The objective of this review is to provide a comprehensive overview of the most reliable and well-validated clinical indications and elucidate the most prominent results of single-pulse TMS-EEG diagnostic and prognostic features among various clinical populations. Our process included a review of the existing data with a common methodology and analytical approach, which indicates putative physiological biomarkers for specific clinical conditions or use.

### 1.1. Single-Pulse TEP

Single-pulse TEP is recorded with interstimulus intervals ranging from 2 to 10 s with or without jitter [3,9]. Stimulation intensity also varies and is usually divided into sub- or supra-motor threshold intensities including 80–90% for sub and 110–120% for supra in most cases [10,11]. TMS potentials evoked by single-pulse stimulations (TEPs) appear as a complex waveform composed of a series of negative and positive peaks and notches at specific latencies that have been consistently characterized, namely N15, P30, N45, P60, N100, P180, and N280 [3,12,13,14,15]. These voltage deflections are thought to represent the response of the stimulated cortical area and propagation of the activity to distant areas [16]. Their latencies and amplitudes have been shown to provide physiological insight into brain excitability and connectivity, and to give an indication of various neurologic and psychiatric conditions [1,3,11].

### 1.2. Quantification of TEP

The main measures representing TMS-EEG response include general response amplitude/area under the curve (AUC) and specific peak latencies and amplitudes. These can be calculated for an average TEP across specific regions of interest (ROIs, a group of specific recording electrodes) or for global mean field potential (GMFP) and local mean field potential/power (LMFP), which reflect the variability in the signal across electrodes as a function of time over the entire surface of the head (GMFP) or in a specific ROI relative to the entire surface (LMFP). These measures are often described as scalp topographical maps that illustrate the distribution of potentials across the electrodes over time. An additional promising computational representation of TEP is termed the perturbational complexity index (PCI), which is an index indicating the complexity of the brain response to TMS. This measure was originally aimed at assessing levels of consciousness but is now used for other indications as well. A low PCI results from reduced integration, or segregation across cortical areas or reduced spatio-stereotypical activity, while a high PCI reflects integrated and differentiated spatio-stereotypical spatiotemporal activations [3].

## 2. Methods

A comprehensive literature search, focused on TMS-EEG studies testing clinically relevant hypotheses including diagnosis of a specific disease or disorder, prognosis, and treatment effect, was conducted. The search was performed in several databases including Pubmed, Embase, and Google Scholar. The following keywords were used in combination: “TMS-EEG”, “TMS evoked potential”, “disease”, ”disorder”, and ”single pulse”; the years of search were limited to 2010–2024, and only original research articles from peer-reviewed journals were included. Results were divided into three main categories: ‘Brain aging and neurodegeneration’, ‘Brain Injury diagnostics and prognostication’, and ‘Neurological and psychiatric treatment response and monitoring’. Within each main category, sub-division was made based on clinical indication (e.g., disease, condition, or application).

This review only included studies utilizing single-pulse TMS-EEG methodology, which spans a frequency range of 0.1 to 0.9 Hz, all considered as single-pulse TMS protocols. No constraints were made on stimulation intensity and site. Stimulation frequency, intensity, and sites are described for each study, along with the study population and main results.

## 3. Review of Results

### 3.1. TEP Diagnostic Utility

Numerous TMS-EEG studies have been performed over the last three decades in multiple clinical groups, including healthy controls, patients with various diseases, and treatment groups. These studies have elucidated consistent trends that may be valuable as a diagnostic aid. Single-pulse TEP has been investigated in healthy and pathological aging, Alzheimer’s disease, Parkinson’s disease, and different brain states, as well as the prediction and monitoring of treatment effects [3].

### 3.2. Brain Aging and Neurodegeneration

#### Healthy Aging

The studies in adult aging populations detailed in Table 1 have indicated a variety of changes in single-pulse TEP that occur with age. We found six different studies examining the changes in single-pulse TMS (spTMS) TEPs in response to M1 (primary motor cortex), DLPFC (dorsolateral prefrontal cortex), angular gyrus, and supramarginal gyrus stimulation in aging populations. These studies showed a consistent decrease in global mean field potential (GMFP) amplitude [17,18] and N45 amplitude [15,17,18], with one study reporting an increase in M1 N45 amplitude [19]. In addition, studies have shown delayed P60 and P180 latencies with aging [15,19,20] and a change in the spatial distribution of N100 and P180 peaks [21]. Both the P60-N100 slope and the N100-P180 slope were shown to decrease with age, which aligns with the general decrease in amplitude and delay in latencies [22]. These results may indicate age-related decreases in excitability and connectivity levels, in line with other modalities and known age-related brain deteriorations. See Table 1 for specific details.

### 3.3. Alzheimer’s Disease

In contrast to the decrease in excitability with age, multiple studies have demonstrated increased excitability in Alzheimer’s disease (AD) patients compared to age-matched controls. This is evident in early features of the TEP response and has been shown consistently in six different studies. These studies indicated an increase in the early amplitude of TEP (ranging from 20 to 90 ms post-stimulation) in response to DLPFC stimulation [14,23,24,25], while two other earlier studies indicated a decrease in P30 amplitude in response to M1L (left primary motor cortex), left superior frontal cortex, and Brodmann’s areas BA6/8 stimulation [26,27]. A longitudinal study that examined the transition from mild cognitive impairment (MCI) to dementia found a decrease in GMFP, in addition to time-specific alterations in global TMS-induced activity [stability of dipolar activity (sDA)], which discriminated npAD-MCI from MCI that will convert to AD [28]. Another study reported a decreased amplitude of TEP and a change in the TEP stereotypical waveform structure of MCI and dementia compared to healthy controls [29].

Additionally, a study that examined later components of TEP (N100, P180) in the classification of AD in response to l-DLPFC (left dorsolateral prefrontal cortex) found that an increase in the LMFP of N100 discriminated AD with an accuracy of 88.37% [30]. See Table 2 for specific details.

### 3.4. Parkinson’s Disease (PD)

Accumulated data from the last few years have TEP to be a promising methodology for treatment monitoring and as a diagnostic aid in Parkinson’s disease (PD) patients (see Table 3 for specific details). In these studies, early TEP component latencies were reported to decrease in PD patients. This decrease was shown to be restored to normal levels with treatment. Three studies focused on TEPs as a diagnostic tool and estimated the neurophysiological differences of PD patients from HCs (healthy controls). One study examined two bilateral targets in M1 and DLPFC and revealed a general change in the TEP stereotypical waveform structure, lower intertrial adherence, decreased left–right interhemispheric connectivity, and lower P60-N100 amplitude [34]. Others showed P30 amplitude was reduced in PD patients in the OFF state compared to HCs and was ‘normalized’ with treatment (ON state) [35]. Later, the same group examined de novo (treatment naïve, early stage) PD patients, and demonstrated lower P30 amplitude compared to controls in response to M1 stimulation [36]. Four studies examined the utility of TEP as an intervention monitoring tool. One study that included 48 PD patients used TEPs to monitor the effect of a multidisciplinary intensive rehabilitation treatment (MIRT), investigated the neurophysiological change measured with M1 TEP, and revealed reduced GMFP amplitudes only in intervention responders [37]. Another study tested the effect of intervention with either combined DBS and levodopa (ON/ON) or DBS (OFF/ON) and no intervention (OFF/OFF). The researchers found a decrease in GMFP amplitude in response to M1 stimulation in the ON states, with only the ON/ON state not significantly lower than HCs [38]. These results were not supported by the findings of another group [39] that tested the differences measured with TEP in the ON versus the OFF state induced by deep brain stimulation (DBS) and yielded no significant findings (though this may be due to small study sample). The fourth study targeted different networks and examined the differences in PD, Parkinson’s disease dementia (PDD), and dementia with Lewy bodies (DLB) patients with and without visual hallucinations (VHs) in response to stimulation of the dorsal attention network (DAN) that included visual areas (right primary and secondary visual cortices, intraparietal sulcus and right frontal eye fields). They found that patients with VHs showed lower TMS-evoked cortical activation within the DAN following intraparietal sulcus and frontal eye field stimulation than patients without VHs [40].

### 3.5. Brain Injury Diagnostic Prognostication

#### Disorders of Consciousness (DOCs)

Disorders of consciousness (DOCs) include unresponsive wakefulness syndrome (UWS), vegetative state (VS), and minimally conscious state (MCS). Earlier studies demonstrated that cortical excitability and connectivity are impaired in DOCs and are manifested by a non-stereotypical, simplified TEP waveform with a lower number of components and decreased spread of the currents induced by the TMS [41,42]. In 2013, a group of investigators introduced the perturbational complexity index (PCI) and demonstrated its reliability and validity in detecting the level of consciousness through the examination of several stimulation targets and intensities in awake and sleeping healthy controls, following the use of anesthetics, and, finally, in VS and MCS patients [43], with another study confirming the effects of anesthetics (propofol, xenon, and ketamine) on PCI [44]. Further TMS-EEG studies have successfully demonstrated the ability of PCI to measure the disruption of information processing and damaged connectivity as well as complexity of the overall brain network, essentially representing an effective connectivity measure in states and disorders of consciousness [45] with replication of results [46]. More recent studies have successfully implemented the PCI^ST^, which demonstrated similar performance to PCI with significantly improved processing times [47]. In a series of clinical studies, PCI measured by TEPs was highly successful in detecting MCS patients and stratifying UWS patients into distinct prognostic categories. There is currently compelling evidence that TMS-EEG is an excellent tool for probing the functional state of thalamocortical circuits and represents a highly promising diagnostic and prognostic biomarker in patients with various unconscious states as has been systematically reviewed [48]. See Table 4 for specific details.

### 3.6. Stroke Rehabilitation and Recovery

In stroke patients, TMS-EEG has been studied mostly to determine prognosis. A simplified TEP waveform, characterized by fewer deflections of distinct peaks, was observed in the injured ipsilateral hemisphere in acute stroke patients [52,53]. Another study showed that the lesioned hemisphere was hyperexcitable compared to healthy controls and the contralesional side [54]. In two studies, reduced M1 N100 amplitude in response to ipsilesional stimulation predicted positive outcome following rehabilitation [55,56]. In subcortical ischemic stroke patients, M1 GMFP was decreased compared to healthy controls; changes in TEP were observed during rehabilitation with an increase in GMFP (50–100 ms) amplitude in the affected hemispheres compared to those unaffected at 40 and 60 days after stroke onset [57]. Additionally, GMFP P30 in response to M1 ipsilesional stimulation was demonstrated to be useful for measuring and monitoring active rTMS intervention compared to sham [58]. See Table 5 for specific details.

### 3.7. Neurological and Psychiatric Treatment Response and Monitoring

#### Major Depressive Disorder (MDD)

**Figure 1:**
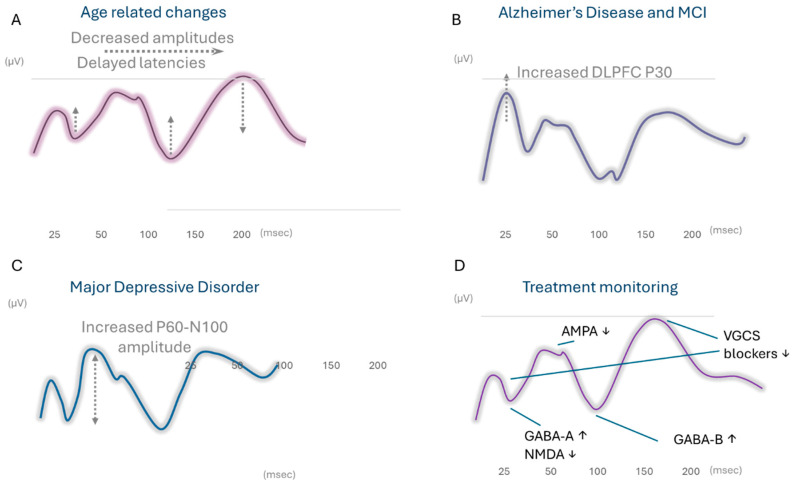
Illustrative examples of the changes in TEP across clinical conditions/interventions. Figure 1—illustrative simulated TEP waveforms showing voltage (μV, y-axis) over time (ms, x-axis). (**A**) Age-related changes showing decreased amplitudes and delayed latency of TEP components. (**B**) Illustration of a typical AD TEP with increased P30. (**C**) Illustration of an MDD TEP waveform with increased baseline P60-N100 amplitude. (**D**) Illustration of the changes in TEP peaks in response to pharmacological interventions.

Patients suffering from depression are believed to have functional and structural abnormalities in DLPFC, such as reduced volume, abnormal activity patterns, or abnormal available connection networks. Common interventions for MDD include pharmacotherapy, psychotherapy, and brain stimulation. Thus, TEP can be a valuable tool for aiding in the diagnostic process, treatment planning, and monitoring. Two large sample-controlled trials in MDD patients reported similar results, indicating decreased excitability levels in MDD patients. In one study, LMFP (local mean field potential) was reduced at latencies around P180 and the P60/N100 ratio was lower in MDD compared to HCs [59]. The second study found that MDD had a smaller P60 amplitude that was associated with more severe depressive symptoms and suggested this to be a marker for the decrease in prefrontal cortical excitability in MDD [60]. See Figure 1C for a waveform simulation illustrating these findings. Additional studies were focused on rTMS (repetitive TMS) effectiveness in the treatment of MDD and are described in the treatment evaluation section. See Table 6 for specific details.

### 3.8. TEP in Prediction and Monitoring of Treatment Response

The use of TEP for the prediction and monitoring of pharmacological and non-pharmacological treatment effects relies on its ability to measure changes in cortical excitability and connectivity in specific brain networks and the association made between specific TEP components and underlying neurotransmission mechanisms. These mechanisms were established in several studies that utilized pharmacological substances and were carried out in healthy participants [61,62,63,64] (See Table 7 for specific details). These studies associated the N45 component with activation of GABA-A (gamma-Aminobutyric acid A receptor) and NMDA (N-methyl-D-aspartate receptor) [65,66,67]; and P60 with AMPA (α-amino-3-hydroxy-5-methyl-4-isoxazole propionic acid receptor) activation [67]. N100 increased with GABA-B (gamma-Aminobutyric acid B receptor) and was reduced with GABA-A activation [65]; P30 increased and P180 decreased with anti-epileptic agents (such as voltage gated sodium channel (VGSC) blockers) [68]. These studies paved the way for the measurement of pharmacological effects on specific neurotransmission mechanisms. See Figure 1D for an illustrative waveform simulation of these effects.

TEP amplitude has also been shown to be decreased with various types of anesthetic agents, suggesting its utility as an index of extent of anesthetic effect [44]. Based on these earlier studies, several more recent randomized controlled trials have used TEPs to investigate the neurophysiological effect of treatment and network neurophysiological status useful in the prediction of treatment response for different conditions. Several studies have demonstrated that in MDD patients treated with rTMS, DLPFC N100 was a useful component for the prediction of treatment response at baseline, with an increase in DLPFC-LMFP (150–185 ms poststimulation) in the treatment arm compared to a sham [59,69]. Further, P60/N100 was effective in diagnosing depression by differentiating it from HCs [59]. Another randomized controlled trial in MDD patients revealed that improvement in depressive symptoms following rTMS treatment correlated with a decrease in DLPFC GMFP-N100 amplitude. A ROC analysis demonstrated that baseline TEP amplitude at the site of rTMS stimulation (left DLPFC) predicted the resolution of suicidality with 87.5% sensitivity, 77.8% specificity, and 82.6% accuracy (AUC = 0.868, *p* = 0.003) [70].

In a randomized controlled trial in early AD patients, Rotigotine (a dopamine agonist) was evaluated as a treatment. DLPFC GMFP was increased in the treatment group compared to placebo [71]. In a randomized controlled cross-over study of epilepsy, the effect of Levetiracetam (used as an adjunct treatment) increased N45 and decreased N100 amplitudes in response to M1 stimulation [72], a pattern previously demonstrated with other anti-seizure medications [67]. TEP measurement has also been demonstrated as useful for determining whether a specific drug successfully crosses the blood–brain barrier (BBB) in phase I studies of healthy controls [66,67,73].

**Table 7:** TEP as measure of treatment response.

Reference	Frequency	Population	Treatment	*n*	Area of Stimulation	Stimulation Intensity	Main Finding
[72]	0.2–0.3	Healthy	Levetiracetam valproic acid lorazepam	16	M1L	120% RMT	Levetiracetam, valproic acid, and lorazepam decreased cortical excitability. Levetiracetam increased TMS-evoked potential component N45 in a central cluster and decreased N100 in a contralateral cluster
[67]	spTMS, not specified	Healthy	Dextromethorphan Perampanel	16	M1L	100%	Dextromethorphan increased the amplitude of the N45 TEP, but it had no effect on TIOs (times of interest). Perampanel reduced P60 TEP amplitude in the non-stimulated hemisphere
[73]	0.4 ± 20%	Healthy	XEN1101 (a novel positive allosteric modulator of the potassium channel- phase I study).	20	M1L	100%	The amplitudes of TEPs occurring at early (15–55 ms after TMS) and at late (150–250 ms after TMS) latencies were significantly suppressed from baseline by 20 mg of XEN1101;
[68]	0.167–0.25	Healthy	BRV CBZ	15	M1	100%	Brivaracetam (BRV; 100 mg) decreased N100; Carbamazepine (CBZ; 600 mg) increased N45.
[74]	0.5 ± 25%	Healthy	DZP	16	M1	70 and 100%	Diazepam (DZP; 20 mg) decreased N100 and P180, and increased N45.
[75]	spTMS, not specified	MDD	rTMS	114	l-LDPFC	120% RMT	Higher baseline N100 predicted treatment success.
[59]	spTMS, not specified	MDD	rTMS	55 MDD 64 HCs	l-DLPFC	100% RMT	P60/N100 and LMFP-AUC (164–215 ms) differentiated MDD from HCs; LMFP-AUC (150–185 ms) changed significantly following treatment compared to sham.
[76]	spTMS, not specified	youth (aged 16–24 years old)	iTBS	20 MDD undergoing (10 iTBS sessions) 30 MDD undergoing 20 iTBS session	l-DLPFC, r-DLPFC, l-IPL, r-IPL	Eliciting a 1 mV motor evoked potentials (MEPs).	Greater (i.e., more negative) N45 and smaller P60 baseline values were associated with greater treatment response to intermittent theta burst stimulation (iTBS).
[70]	spTMS, not specified	MDD	rTMS	30	l-DLPFC	peak-to-peak MEP amplitude of 1 mV in 20 trials	N45 and N100 amplitudes (t = 2.177, *p* = 0.042) decreased after active rTMS. GMFP N100 amplitude decreased with improvement in depressive symptoms. ROC analysis demonstrated that baseline TEP amplitude at site of rTMS stimulation (left DLPFC) predicted resolution of suicidality with 87.5% sensitivity, 77.8% specificity, and 82.6% accuracy (AUC = 0.868, *p* = 0.003)
[77]	spTMS, not specified	Adolescent MDD	rTMS	36	l-DLPFC and r-DLPFC	120% RMT	N100 amplitude significantly decreased in response to intervention (emotional 1-back task and paired-pulse protocol applied at M1.) l-DLPFC N100 amplitude (but not r-DLPFC) was negatively correlated with depression severity.
[71]	spTMS, not specified	AD	Rotigotine	43 (Rotigotine) 43 (Placebo)	left DLPFC and left PPC	80% RMT	DLPFC GMFP was increased in group treated with Rotigotine (dopamine agonist) compared with placebo.

GMFP—global mean field power; HCs—healthy controls; M1L—left primary motor cortex; RMT—rest motor threshold; spTMS—single-pulse TMS.

## 4. Discussion

**Figure 2:**
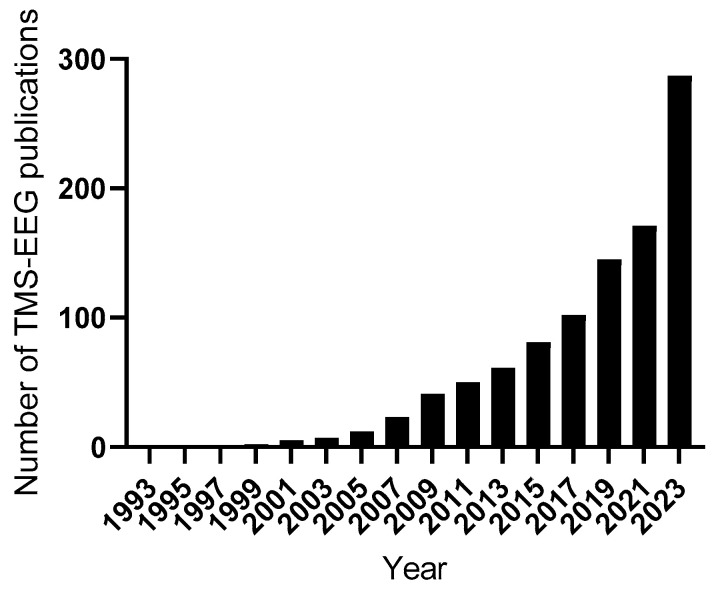
Rise in TMS-EEG publications from 1993 to 2023. Bars represent the number of publications on TMS-EEG each year.

TMS-EEG is a powerful non-invasive and safe tool that can provide direct physiological insights to the physician in routine clinical care and aid in the diagnosis, monitoring, and prognostication of disease and treatment. Substantial evidence has accumulated to date regarding TEPs in various clinical indications, with exponential growth in the number of studies published every year (Figure 2).

One of the most well-studied indications relates to the diagnosis of AD, in which early TEP (<80 ms) hyperexcitability was repeatedly reported in response to DLPFC stimulation (Figure 1B—illustrative simulation of TEP). This aligns with previous meta-analyses pointing to a decreased RMT in the early stages of disease; specifically, a decreased RMT in the early stage of AD reflects an increase in excitability or excitatory/inhibitory balance, possibly attributable to the degeneration of and decline in inhibitory interneuron function in M1 [78]. Interestingly, findings in healthy aging reflect an overall decrease in TEP amplitudes (Figure 1A—illustrative simulation of TEP), indicating decreased cortical excitability with age. These studies highlight TMS-EEG as a potential tool for distinguishing normal healthy aging from abnormal aging. Another neurodegenerative disease with a growing body of evidence is PD, for which TEP has been shown useful for monitoring levodopa or DBS treatment effect. In these studies, P30/early latencies were reported to be reduced in PD patients but to reach normal levels with treatment

TMS-EEG has also shown great promise in monitoring disorders of consciousness (DOCs) following acute brain injury [79]. The monitoring and prognosis of DOCs by evaluation of TEP complexity has already been established and validated, with specific thresholds for DOC states. An additional substantial body of evidence has been gathered around possible markers for diagnosis of major depressive disorder (MDD) (see Figure 1C for an illustrative simulation of TEP), and prefrontal cortical excitability seems linked to the severity of depressive symptoms, with the use of TEPs as direct measurements for the monitoring and prediction of response to treatment with rTMS. Several large randomized controlled studies have been conducted and underscore the value of TEPs in detecting brain physiological changes associated with pharmacological agents affecting the excitation/inhibition balance, as well as other interventions like rTMS. In stroke recovery and rehabilitation, evidence is accumulating for characteristic changes in TEP in the acute stage as well as the utility of TEP as a prognostic measure for recovery. Notably, alterations in cortical excitability are considered core to the disease in focal and generalized epilepsies [80,81]. Thus, examining cortical excitability may yield TEP markers to aid in the process of epilepsy diagnosis and to monitor response to anti-seizure medication. Several studies have utilized single-pulse TEP to monitor treatment response to anti-seizure medications. In these studies reviewed in [82], an increased N45, and decreased N100 and P180 were most often reported for one dose of an anti-seizure medication (N45 8/15 studies, N100 7/15, and P180 6/15) (see Figure 1D—illustrative simulation of TEP).

In this review, we sought to elucidate the clinical indications that show the best established and repeatable results to date. We focused our search on single-pulse TMS-EEG studies that analyzed TEPs in clinically relevant indications. TMS-EEG is used for a wide variety of neurological and psychiatric conditions ranging from stroke, traumatic brain injuries (TBIs), and neurodegenerative disorders like dementia and amyotrophic lateral sclerosis (ALS) to schizophrenia, bipolar disorder, and attention deficit hyperactivity disorder (ADHD). Of the studied indications that show promising results, several indications discussed above stand out.

The main parameters of the TEP methodology include stimulation intensity, frequency, and stimulation site. Our review specifically focused on single-pulse TEP methodology, which is commonly used for evaluating the baseline-evoked response to TMS stimulation. Single-pulse TMS methodology includes frequencies ranging from 0.1 Hz to 0.9 Hz, which are considered equivalent. Stimulation sites were usually selected in accord with the clinical condition, putative mechanisms or pathology. No clear conclusion can be drawn regarding stimulation intensity. Across the clinical indications described in this review, there were no common trends with respect to specific intensities.

This review demonstrates the applicability of single-pulse TEP in the clinical setting. It is evident that this methodology holds great promise for early implementation in clinical practice, leading to improved brain health and disease management.

## 5. Limitations

TMS-EEG methodology is heterogeneous, with different intensities and stimulation sites used across studies. This review presents the results of a comprehensive search for studies utilizing single-pulse TMS-EEG. Though there are some consistent studies in specific clinical indications, these results are not sufficiently robust. In addition, in view of calls for standardized methodology and the study of larger samples, this field is rapidly evolving. Additional randomized studies with a standardized methodology are required to further establish single-pulse TMS-EEG for clinical use.

## Figures and Tables

**Table 1:** Healthy Aging.

Study	Frequency (Hz)	Younger (Mean Age ± std)	Older (Mean Age ± std)	Area of Stimulation	Stimulation Intensity	Main Finding
[20]	0.2–0.25	21 (28.1 ± 3.2)	21 (62.8 ± 4.2)	M1L	110% RMT	N100 amplitudes were significantly reduced in older participants. N100 and P180 latencies were significantly delayed in older participants. Results did not indicate a link between the interhemispheric differences in TEP and age.
[17]	0.25–0.5	21 (22.6 ± 2.6)	20 (67 ± 3.11)	Angular gyrus/supramarginal gyrus	80% RMT	Decreased GMFP amplitude within 100–200 ms in older compared to young adults. Older adults showed smaller N45 amplitude and delayed P180 latency.
[15]	Single (not specified)	12 (39 ± 12)	12 (72 ± 9)	M1L, DLPFC	1 mV p2p	M1L: Reduced amplitudes of N45 and P180 and delayed latency of P60 in older adults. Left DLPFC stimulation revealed: Decreased N45 amplitude and delayed latency in N45-P60 over the right central region in older adults.
[22]	0.1, 1	30 (35 ± 6.6)	30 (61 ± 5.9), 17 (75.4 ± 5.6)	M1L	25–60% DI	P60-N100 slope, N100-P180 slope decreased with age. General and late latency STP (relative amplitude of response to 1 Hz stimulation versus spTMS) was increased.
[19]	0.2	17 (24.2 ± 1)	17 (71.4 ± 1)	M1L	120% RMT	N45 amplitude was increased in older adults, and both N100 and P180 showed altered spatial distributions. Earlier P30 and later P180 were observed in the elderly group.
[18]	0.167–0.25	12 (24.5)	12 (67.6)	M1L	120% RMT	GMFP decreased with age. P30 was globally increased in the older participants. N45 was decreased ipsilateral to stimulation. N100 was decreased (frontally) and increased (at Cz) and P180 was globally decreased in older subjects.

DI—device maximal intensity; GMFP—global mean field power; l-DLPFC—left dorsolateral prefrontal cortex; M1L—left primary motor cortex; RMT—rest motor threshold; spTMS—single-pulse TMS.

**Table 2:** Alzheimer’s disease (AD).

Reference	Frequency	AD	HCs	Area of Stimulation	Stimulation Intensity	Main Finding
[31]	0.25–0.5	38	17	l-DLPFC	90% MT	Classification with random forest analysis. Increased 45–80 ms amplitude in AD.
[25]	0.25–0.5	65	21	l-DLPFC, PC, l-PPC	90% MT	Hyperexcitability of AD compared to controls in the precuneus (PC) (P30, P65, P120) and DLPFC (increased P30 amplitude). PC was correlated to MMSE score and CSF aϐ, while PCC P30 was correlated with Tau and p-Tau levels.
[24]	0.25–0.5	17	17	l-DLPFC	90% MT	Increase in 25–40, 45–80, 85–150, 160–250 amplitudes and GMFP.
[32]	Not specified.	3 AD	8	Vertex	120% MT	People with severe AD demonstrated a PCI^ST^ near or below the threshold for consciousness.
[23]	0.1	24	11	DLPFC	SI-1mv	Increased amplitude in 25–80 ms (no difference in P30, N45, P60).
[29]	0.1	17 Subjective cognitive decline (SCD) 12 amnestic mild cognitive impairment (aMCI) 11 Dementia	15	M1L	80% MT	Lower amplitude (AUC) of entire TEP and a change in TEP stereotypical waveform structure of subjective cognitive decline, MCI, and mild dementia groups compared to cognitively normal controls. STP (relative amplitude of response to 1 Hz stimulation versus spTMS) was different only in aMCI and dementia but not in SCD.
[30]	0.3	21 (Cognitive Impairment (CI)	22	l-DLPFC	120% RMT	M100 (LMFP), N100 average value (AVG), N100 latency and amplitude contributed most in classification of AD
[28]	0.125–0.167	17 aMCI (7 pMCI turn over to AD in the course of 6 years FU, 6 npMCI- no turn over to AD).	15	M1L	120% RMT	Stability of dipolar activity (sDA)-STD of GMFP across time was reduced in pMCI compared with npAD-MCI.
[33]	0.25–0.5	34		l-DLPFC	110% MT	P30 amplitude increased as MMSE decreased (significant negative correlation of P30 and cognitive score in MMSE)
[22]	0.1, 1	20 (75.2)	17	M1L	25–60% of Device Intensity	P60-N100 slope, N100-P180 slope, general and late latencies were shorter and STP (relative amplitude of response to 1 Hz stimulation versus spTMS) was decreased in mild dementia compared to age-matched controls.
[14]	0.125–0.167	12	12	M1L	120% MT	Higher GMFP 24–90 ms, higher P30 and P60 amplitudes, and delayed latency of N100 in AD compared to controls.
[26]	0.3–0.5	5 AD 5 MCI	4	M1L, M1R	110% MT	Decreased P30 amplitude in MCI and AD compared to controls, with successful discrimination of HCs from MCI and AD and an inverse correlation with global CDR and CDR-SOB.
[27]	0.5–0.6	9 AD	9 elder healthy 9 young healthy	left superior frontal cortex (Brodmann’s areas BA6/8)	110 V/m	Amplitude of early TEP latencies (10–45 ms) was reduced in AD compared to controls.

AD—Alzheimer’s disease; DI—device maximal intensity; GMFP—global mean field power; HCs—healthy controls; PCI^ST^—fast perturbational complexity index, l-DLPFC—left dorsolateral prefrontal cortex; M1L—primary motor cortex; MMSE—mini mental state examination; PC—precuneus; l-PPC—left posterior parietal cortex; RMT—rest motor threshold; spTMS—single-pulse TMS.

**Table 3:** Parkinson’s disease (PD).

Reference	Frequency	PD	HCs	Area of Stimulation	Stimulation Intensity	Main Finding
[36]	spMT (not specified)	28 early, drug-naive PD	28	M1 or pre-SMA	110% RMT	Reduced M1 TEP P30 amplitude in de novo PD patients compared to HCs and similar pre-SMA TEP N40 amplitude between groups.
[40]	0.2 Hz	12 PD 8 PDD 6 DLB (11 had VHs and 15 did not have VHs)	-	Right V1, right V2, intraparietal sulcus, and the right frontal eye fields	160% RMT	Patients with VHs showed decreased TMS-evoked cortical activation within the DAN relative to patients without VHs following intraparietal sulcus and frontal eye field stimulation. No difference was found between patients with and without cognitive impairment.
[39]	0.2–0.33 Hz	6 (PD with motor fluctuations)	-	M1 Pre-supplementary motor area (pre-SMA) Inferior frontal gyrus (IFG)	120% RMT	No significant treatment effects observed. Tendency of LMFP measured in response to IFG stimulation to show the strongest difference between the ON and OFF DBS conditions, with higher LMFP at OFF state in 60–100 ms. M1 LMFP differences between ON/OFF were in 10 to 30 ms TEP latencies.
[37]	0.25–0.5 Hz	48	-	M1L	90% RMT	Primary motor cortex stimulation reduced GMFP amplitudes in responders but not significantly in non-responders (multidisciplinary intensive rehabilitation treatment (MIRT)).
[36]	0.6 Hz	20 (PD with motor fluctuations)	19	M1 and pre-SMA	110% RMT	Compared to HCs, PD (OFF) patients had smaller P30 responses from the M1s contralateral (M1+) and ipsilateral (M1−) to the most bradykinetic side and increased pre-SMA N40. Dopaminergic therapy normalized the amplitude of M1+ and M1− P30 as well as pre-SMA N40. Positive correlation between M1+ P30 amplitude and bradykinesia in PD (OFF) patients.
[34]	0.1 Hz	32	21	M1L, M1R, l-DLPFC, r-DLPFC	80% RMT	PD TEP stereotypical waveform structure changes, lower intertrial adherence, decreased left–right interhemispheric connectivity, and lower P60-N100 amplitude.
[38]	0.16–0.25 Hz	6 (advanced akinetic–rigid PD)	8	M1L	90% RMT	A significant increase in GMFP amplitude in DBS (OFF/ON) only vs. no intervention (OFF/OFF) from 63–80 ms after the TMS pulse. Both levodopa and DBS (ON/ON) compared to no intervention (OFF/OFF) revealed a significant increase in GMFP from 70–80 ms after TMS and from 108–128 ms after TMS. Both levodopa and DBS (ON/ON) compared to only DBS condition (OFF/ON) showed a significant increase in GMFP from 107–147 ms after TMS. Only the ON/ON state was not reduced relative to HCs.

DAN—dorsal attention network (DAN); DBS—deep brain stimulation; l-DLPFC—left dorsolateral prefrontal cortex; r-DLPFC—right dorsolateral prefrontal cortex; GMFP—global mean field power; HCs—healthy controls; M1L—left primary motor cortex; pre-SMA—pre-supplementary motor area; RMT—rest motor threshold; V1—primary visual cortex; V2—secondary visual cortex; VHs—visual hallucinations.

**Table 4:** Disorders of consciousness (DOCs).

Reference	Freq	DOCs	HCs	Area of Stimulation	Stimulation Intensity	Main Finding
[49]	0.4 Hz	48 (28 MCS, 20 UWS)	25	Frontal, senso-motor, parietal	90% MT	Reduced spatial and temporal propagation. PSD, oscillations.
[46]	0.43–0.5 Hz	12 MCS 11 UWS	-	Superior frontal and/or parietal cortex	120 V/m	Successfully replicated the performance of PCI in discriminating between UWS and MCS patients of Casarotto et al. [45]
[47]	Single, not specified	49 MCS 43 UWS 5 LIS 11 EMCS	108	Middle-caudal portion of the superior frontal gyrus; and superior parietal lobule	120 V/m −160 V/m	Introduced PCI^ST^, a faster method for estimating perturbational complexity and demonstrated same accuracy as the original PCI.
[50]	Single, not specified	15 MCS 3 UWS 3 EMCS	14	Left or right superior parietal lobule and superior frontal lobule	100–150 V/m	Global FA predicted 74% of PCI variance in the whole sample and 56% in the patient group. No other predictors (age, gender, time since onset, behavioral score) improved the models. FA and PCI were correlated in the whole population (r = 0.86, *p* < 0.0001), as well as in the patient and healthy subgroups.
[51]	0.43–0.5 Hz	11 MCS 9 UWS 2 EMCS 2 LIS	-	Left and right medial part of the superior frontal and parietal gyri	120 V/m	FDG-PET and PCI showed congruent results in 22 patients, regardless of their behavioral diagnosis. Notably, FDG-PET and PCI revealed preserved metabolic rates and high complexity levels in four patients who were behaviorally unresponsive.
[45]	Single, not specified	43 MCS 38 VS	150 healthy different states (sleep and anesthesia) and conscious injured patient (benchmark population)	Middle-caudal portion of the superior frontal gyrus; and superior parietal lobule	120 V/m −160 V/m	Established PCI threshold (0.31) in a benchmark population. In a validation cohort of MCS and VS max PCI classified MCS with high sensitivity. VS patients demonstrated 3 levels of complexity. Concerning the outcome at 6 months, 6 of 9 (1 unknown) high-complexity VS patients transitioned to a behavioral MCS, whereas such a transition was observed in 5 of 21 (2 unknown) low-complexity patients. None of the no-response subgroup showed improvement.
[44]	0.5 Hz	-	18 HCs (randomly assigned to propofol, xenon, and ketamine)	Superior parietal gyrus (BA07), the rostral portion of the premotor cortex (BA06)	110 V/m	Complexity of EEG responses was high during wakefulness, low when subjects reported no conscious experiences upon emergence from anesthesia (propofol and xenon), and high when they reported intense dreams (ketamine).
[42]	0.25–0.5 Hz	5 MCS 8 VS	5	M1L or M1R	75% DI	VS patients exhibited no or only ipsilateral TEPs with reduced amplitudes.
[43]	not specified	6 VS/UWS 2 LIS 6 MCS 6 EMCS	32 HCs	Superior occipital gyrus (BA19), the middle superior frontal gyrus (BA08), the superior parietal gyrus (BA07), the rostral portion of the premotor cortex (BA06), and the midline sensorimotor cortex (BA04)	90–160 V/m	Data collected from previous experiments were analyzed and introduced in support of the reliability and validity of the PCI index for measurements of consciousness. Awake healthy subject PCI values ranged from 0.44 to 0.67 and decreased to 0.18–0.28 during NREM sleep. PCI also decreased with use of anesthetics with midazolam deep sedation (0.23 to 0.31), propofol (0.13–0.30), and xenon (0.12–0.31). In DOC patients, minimally conscious state showed intermediate values (0.32–0.49), vegetative state being unconscious with lowest values(0.19–0.31) and those with locked-in syndrome clearly aware showing highest values (0.51–0.62). Conscious state was reflected in the PCI of a VS patient that recovered a minimal level of consciousness.
[41]	0.4–0.5 Hz	5 MCS 5 V 2 LIS	-	the left and right medial third of the superior parietal gyrus and the left and right medial third of the superior frontal gyrus.	140 V/m −160 V/m	GMFPs of VS remained localized involving a small number of sources around the stimulated area.

DI—device maximal intensity; PSD—power spectral density; EMCS—emergence from the minimally conscious state; FA—fractional anisotropy measured in MRI-DTI; GMFP—global mean field power; HCs—healthy controls; LIS—locked-in syndrome; M1L—primary motor cortex; MCS—minimally conscious state; NREM—nonrapid eye movement; UWS—unresponsive wakefulness syndrome, VS—vegetative state.

**Table 5:** Stroke.

Study	Freq	Stroke	HCs	Area of Stimulation	Stimulation Intensity	Main Finding
[54]	0.16–0.25	17	9	M1L, M1R	120% RMT	Lesioned hemisphere hyperexcitable compared to healthy controls and the contralesional side.
[58]	spTMS, Not specified	20	-	Ipsilesional M1	120% RMT	GMFP P30 (ipsilesional M1) increased following active rTMS treatment but not following sham.
[56]	0.2–0.25	23	21	Ipsilesional M1	120% RMT	Reduction in N100 amplitude around the stimulated M1, which also correlated with MEP amplitude and RMT.
[53]	0.125–0.16	28	-	Ipsilesional M1	80% RMT	Simplified TEPs with fewer components/peaks ipsilateral to the lesion compared to contralateral hemisphere.
[52]	0.5 Hz	30	-	Ipsilesional M1	electric field of ~120 V/m	Perilesional stimulation resulted in simpler TEPs.
[57]	0.25–0.5	13	10	Ipsilesional M1	90% RMT	M1 GMFP decreased compared to controls. Increased GMFP (50–100 ms) interhemispheric amplitude differences with longer time from cerebrovascular event.
[55]	0.2–0.25	9	-	Ipsilesional M1	110% RMT	Reduced M1 N100 amplitude predicted positive rehabilitation outcome.

GMFP—global mean field power; HCs—healthy controls; M1L—left primary motor cortex; M1R—right primary motor cortex; RMT—rest motor threshold; spTMS—single-pulse TMS.

**Table 6:** Major depressive disorder (MDD).

Reference	Frequency	MDD	HCs	Area of Stimulation	Stimulation Intensity	Main Finding
[59]	spTMS, not specified	74	64	l-DLPFC (F3)	100% RMT	LMFP (164–215 ms) was reduced in MDD and P60/N100 ratio lower compared to controls.
[60]	0.2 Hz	42	41	l-DLPFC(F3)	100% RMT	P60 smaller for MDD and associated with increase in depressive symptoms.

l-DLPFC—left dorsolateral prefrontal cortex; spTMS—single-pulse TMS.
